# Assessing the Potential of Nutraceuticals as Geroprotectors on Muscle Performance and Cognition in Aging Mice

**DOI:** 10.3390/antiox10091415

**Published:** 2021-09-04

**Authors:** Zoltán Singlár, Péter Szentesi, János Fodor, Ágnes Angyal, László Csernoch, Mónika Sztretye

**Affiliations:** 1 Department of Physiology, Faculty of Medicine, University of Debrecen, 4032 Debrecen, Hungary; singlar.zoltan@med.unideb.hu (Z.S.); szentesi.peter@med.unideb.hu (P.S.); fodor.janos@med.unideb.hu (J.F.); angyal.agnes@med.unideb.hu (Á.A.); csl@edu.unideb.hu (L.C.); 2 Doctoral School of Molecular Medicine, University of Debrecen, 4032 Debrecen, Hungary

**Keywords:** skeletal muscle, calcium homeostasis, muscle performance, excitation-contraction coupling, astaxanthin, krill oil, nutraceutical, cognition, mitochondrial calcium, aging

## Abstract

Aging and frailty are associated with a decline in muscle force generation, which is a direct consequence of reduced muscle quantity and quality. Among the leading contributors to aging is the generation of reactive oxygen species, the byproducts of terminal oxidation. Their negative effects can be moderated via antioxidant supplementation. Krill oil and astaxanthin (AX) are nutraceuticals with a variety of health promoting, geroprotective, anti-inflammatory, anti-diabetic and anti-fatigue effects. In this work, we examined the functional effects of these two nutraceutical agents supplemented via pelleted chow in aging mice by examining in vivo and in vitro skeletal muscle function, along with aspects of intracellular and mitochondrial calcium homeostasis, as well as cognition and spatial memory. AX diet regimen limited weight gain compared to the control group; however, this phenomenon was not accompanied by muscle tissue mass decline. On the other hand, both AX and krill oil supplementation increased force production without altering calcium homeostasis during excitation-contraction coupling mechanism or mitochondrial calcium uptake processes. We also provide evidence of improved spatial memory and learning ability in aging mice because of krill oil supplementation. Taken together, our data favors the application of antioxidant nutraceuticals as geroprotectors to improve cognition and healthy aging by virtue of improved skeletal muscle force production.

## 1. Introduction

Aging is a physiological process causing a general receding of physical and mental capacities. Skeletal muscle is highly affected as there is a gradual loss of muscle mass and function (sarcopenia), fatigue/exhaustion, weakness and mobility deterioration (frailty) which impairs the quality of life in elderly people [1]. Nowadays an increasing proportion of the global population is of advanced age and this is predicted to triple between 2017 and 2050 [2]. Despite numerous studies, the mechanism of aging remains elusive. To date, several theories were proposed to explain it such as genetic predisposition, programmed senescence, DNA deterioration, endocrine malfunction, the free radical rationale and mitochondrial dysfunction [3,4,5]. Understanding the cellular mechanism of aging and its pathogenesis are essential tasks for the development of prophylactic and therapeutic strategies to ensure healthy aging and improve the life quality of those affected.

Multiple lines of evidence denote that the mitochondrion, this highly dynamic organelle due to its multiple cellular functions, holds a pivotal role in aging and age-related pathologies. Mitochondria generate cellular energy (ATP), produce reactive oxygen species (ROS) that govern physiological processes [6] and are actively involved in the control of cell death [7]. Physical frailty in the elderly is marked by mitochondrial malfunction in skeletal muscles associated with accumulation of mitochondrial damage, increased oxidative stress, mitochondrial DNA depletion and ultimately cellular senescence [1,8,9]. Mitochondrial network dynamics are tightly controlled through the delicate balance between fusion and fission events. Mitofusin 1 and 2 (Mfn1/2) participate in the outer mitochondrial membrane (OMM) fusion while optic atrophy 1 (OPA1) mediates fusion of the inner mitochondrial membrane (IMM). Fission of mitochondria is controlled by mitochondrial fission 1 (Fis1), dynamin-related protein 1 (Drp1) and mitochondrial fission factor (Mff) proteins [10]. Mfn2 is present in both mitochondria and endoplasmic (sarcoplasmic) reticulum (ER/SR) membranes of skeletal muscles and it has been shown to play a key role in mitochondrial activities such as morphology, localization, turnover, trafficking and functional SR-mitochondrial Ca^2+^ crosstalk [11,12,13]. Aging is associated with a continuous reduction in Mfn2 levels and consequently the Mfn2 deficiency was found to deteriorate mitophagy and increase oxidative stress, ultimately leading to the accumulation of defective mitochondria in mouse skeletal muscles [14]. Furthermore, Mfn2 tethers mitochondria to the SR, facilitating an efficient local mitochondrial Ca^2+^ uptake via the calcium selective channel of the IMM: the mitochondrial Ca^2+^ uniporter (mtCU) [15]. Mitochondrial Ca^2+^ uptake 1 (MICU1) was described initially in 2010 as a regulator of the channel [16]. In 2011, in two independent studies the groups of Mootha and Rizzuto [17,18] discovered a two transmembrane domain containing protein as the molecular identity of the mtCU pore and later this entity was termed as MCU (mitochondrial calcium uniporter) [19]. Recently, studies have shed light on the role of the endocannabinoid system as well, namely cannabinoid receptors 1 (CB1) as regulators of mitochondrial oxidative activity [20]. Mitochondrial CB1 receptors (mtCB1Rs) are found in neurons of the central nervous system (CNS) and in similar proportions in the peripheral tissues such as striated muscles where they are located in the OMM [21]. The activation of the mtCB1Rs has been proposed to be involved in the regulation of oxidative activity of the muscle possibly through the related enzymes implicated in the pyruvate metabolism, a main constituent for the Krebs cycle activity.

Nowadays, researchers agree that disturbances of cellular calcium homeostasis contribute to aging, more specifically in excitable cells such as skeletal muscles and neurons. excitation-contraction coupling (ECC) is a physiological process linking electrical excitation of muscles via the CNS to mechanical contraction [22]. It was proposed that a decline in the Ca^2+^ ion supply available for generating muscle contraction may be one of the pivotal aspects in explaining age-related muscle weakness [23,24,25]. Aging impairs calcium communication between SR and mitochondria which otherwise are tightly interconnected in striated muscles [26,27] and these events can potentially contribute to the decline of muscle performance observed in aging [28]. Pietrangelo and coworkers described an age-related structural disconnection between calcium release units and mitochondria in muscle which alters the specific control of intracellular calcium levels and therefore the efficiency of ATP synthesis processes [29].

Hindered calcium homeostasis, muscle weakness along with elevated oxidative stress, oxidative alterations of proteins, apoptosis and decline of cognition are all unfortunate features of aging. While it seems that oxidative stress is crucial for the progression of aging, no standard therapy to overcome it has been established so far. Many geroprotector agents [30] and various methods have been proposed and tested to handle anti-aging, such as caloric restriction [31], hormonal therapies [32], telomere-based therapies [33], stem cell therapies [34] and antioxidant supplementation [35]. Antioxidants are promising tools that are proposed to induce antioxidant gene expression and provide anti-apoptotic protection of various organs [36]. Astaxanthin (AX) is a naturally occurring xanthophyll carotenoid described in various organisms, such as microalgae, crustaceans and fish [37]. AX is fat soluble and has emerged from use as a food coloring to a highly promising anti-aging geroprotective compound [37] that was reported to be a 100–500 times more powerful antioxidant than vitamin E [38], effectively scavenging free radicals, quenching singlet oxygen, enhancing antioxidant activities, reducing oxidative stress and improving muscle and cognitive function and neural plasticity in mice [38,39,40]. The nutraceutical application of AX for human consumption in doses between 6 and 8 mg/day is widely accepted and well tolerated without any safety concerns [41]. Krill has emerged as a novel sustainable source of omega-3 polyunsaturated fatty acids (n-3 PUFAs); its oil form is remarkably rich in the long-chain PUFAs eicosapentaenoic acid (EPA) and docosahexaenoic acid (DHA) that are essential fatty acids for basic brain function [42]. Moreover, krill oil contains bioactive ingredients such as AX, choline and essential amino acids which cannot be synthesized endogenously; nonetheless, krill oil has been described as extremely beneficial to brain fitness, cognition and memory [43,44]. The idea of krill potentially influencing metabolism, behavior and inflammatory processes through the endocannabinoid system has also emerged [45]. Thus, finding a way to prevent and/or reverse neuro-inflammation with the associated cognitive decline becomes increasingly of pivotal social importance in the context of aging.

In the present work, we have investigated the molecular mechanism by which AX and krill oil supplementation influences skeletal muscle performance and exerts favorable effects on mitochondrial and cognitive function in aging mice. We propose that the administration of geroprotector nutraceuticals such as AX and krill oil would ameliorate the progression of aging by suppressing aging-induced muscle fitness loss and the subsequent cognition impairments. Due to its high-energy metabolism, both the human brain and the skeletal muscles are especially vulnerable to oxidative stress. Here, we hypothesized that krill oil being able to readily cross the blood–brain barrier and with its unique content of PUFAs could potentially counteract the negative effects of excessive ROS production and improve cognition. Our results support the positive health outcomes of nutraceutical administration as we observed improved cognition and skeletal muscle function without major alterations of the excitation-contraction coupling and mitochondrial dynamics.

## 2. Materials and Methods

### 2.1. Animals

Thirty-two C57BL/6 mixed gender mice were used in this study between 13 and 17 months of age. Mice were anaesthetized and sacrificed following an approved protocol by the institutional Animal Care Committee of the University of Debrecen (3-1/2019/DE MAB). Animals were housed in a germ-free environment in mesh covered plastic cages and had *ad libitum* access to water and pelleted mouse chow. Room illumination was on an automated cycle of 12 h dark and 12 h light and the temperature was between 22 and 25 °C. After CO_2_ overdose and manual cervical dislocation, *m. flexor digitorum brevis* (FDB), *m. extensor digitorum longus* (EDL) from the hind limb, *m. tibialis anterior* (TA) and *glutealis* muscles were dissected manually.

### 2.2. Nutraceutical Diet Regimen

Both AX and krill oil diet lasted four weeks while the littermates were fed with standard rodent chow (CTRL group). The AX supplemented special chow was prepared by adding 4 g/kg of AstaReal A1010 (AstaReal Co., Ltd., Nacka, Sweden) dissolved in 100% ethanol to the standard rodent pellet for a final concentration of 0.02% AX. To achieve similar concentration of AX in the krill oil supplemented group we used 25 g/kg of krill oil supplied by Rimfrost Inc. (Alesund, Norway).

### 2.3. In Vivo Experiments

#### 2.3.1. Body Weight Measurement

For each individual mouse in CTRL, AX and krill oil group we measured the body weight before starting the nutraceutical diet (day 1) and after the four weeks feeding period (day 28). The change of body weight along with food consumption were monitored, recorded and averaged by groups.

#### 2.3.2. Forepaw Grip Test

The force of forepaw was monitored before and following four weeks of nutraceutical feeding period. The procedure is described in detail in our earlier report [46].

#### 2.3.3. Barnes–Maze Protocol

In order to evaluate the effect of krill oil supplementation on spatial learning and memory of old mice, the basics of the Barnes–Maze Protocol [47] were applied. The test was held on a 92 cm circular wooden platform, with 20 holes (5 cm diameter) equally distributed along the edge (Supplementary Figure S1). Under one specific hole a plastic goal box filled with litter was mounted. The platform was placed to be surrounded by three distinct visual clues. Given the specificity of the hole with the goal box, the platform was strictly kept in place during the whole procedure. The platform was well illuminated with white light from above and video recordings were taken from an overhead web camera (Genius WideCam F100, Genius, New Taipei City, Taiwan) connected to a notebook with Windows operating system. The protocol can be divided into three parts. During habituation (day 1), each mouse was placed in the middle of the apparatus and covered with a starting cylinder (8 cm diameter) for 1 min. After removal of the cylinder the mouse was allowed to familiarize itself with the surroundings for 5 min or until they found and entered the escape box. The acquisition training was held for 10 days, twice a day per mouse with 1-hour intervals. The mouse was covered with the starting cylinder for 15 s then after its removal the mouse was free to find the position of the escape box within 3 min. To drive the mice to search, 90 dB white background noise was used. If the mice failed to find the escape hole in time, they were placed in the box and were allowed to stay there for 1 min for familiarization purposes. The probe trial was held 3 days after the acquisition training. All the steps were the same except the length of the test was 1 min and the escape box was removed.

#### 2.3.4. Video Tracking Using Animal Tracker

We used AnimalTracker [48] which is an open-source plugin in ImageJ (NIH, Bethesda, MD, USA) image processing program to support animal behavioral analyses. We adapted its Water–Maze module to Barnes–Maze to calculate the time and distance covered by the mice from the starting position to the escape box. Two representative supplementary videos (Control.mp4 and Krill.mp4) show the movement of mice on the sixth day of training protocol. Corresponding to this we included a supplementary image (Supplementary Figure S1) to show the result of animal tracking by the AnimalTracker plugin.

### 2.4. In Vitro Experiments

#### 2.4.1. Measurement of EDL Muscle Force

Muscle contractions were measured similarly as described in our earlier reports [39,49].

#### 2.4.2. Isolating Single FDB Muscle Fibers

For all calcium measurements we used single skeletal muscle fibers obtained from enzymatically digested FDB muscles of the mice. Following manual dissection of the FDB muscles in normal Tyrode’s solution, enzymatic dissociation was done in calcium free Tyrode’s solution which included 0.2% Type I collagenase (Sigma-Aldrich, St. Louis, MO, USA, cat. no. SCR103). The incubation time was 50 min at 37 °C. After the enzymatic treatment FDB muscles were placed in normal Tyrode’s solution and stored in the refrigerator at 4 °C for up to 36 h until use [50,51].

#### 2.4.3. Voltage Clamp

The detailed design of the experimental setup is described in our earlier reports [39,52]. To describe the voltage (V_m_) dependence of calcium transient’s activation, the following Boltzmann function was used:

Ca (V) = Ca_max_/(1 + exp(−(V_m_ − V_50_))/k) (1)

in which the value of V_50_ (the transition voltage) and 1/k (the limiting logarithmic slope) were of interest for us. Individual data points were normalized to Ca_max_ and plotted as a function of the membrane potential to display the voltage dependence of the activation.

#### 2.4.4. Mitochondrial Calcium Uptake

Following Ainbinder and coworkers [12], we recorded the changes in mitochondrial calcium levels in single FDB fibers following repetitive tetanic stimulation using a confocal microscope (Zeiss 5 Live, Oberkochen, Germany). The FDBs were loaded with 5 μM rhod-2-AM for 15 min at room temperature and then washed with dye free normal Tyrode’s solution. Using a pair of platinum electrodes placed close to the fiber of interest, we applied a single or a series of five consecutive tetani (500 ms duration, 100 Hz) at close to supramaximal activating voltage (S88 Stimulator, Grass Technologies, Warwick, RI, USA).

#### 2.4.5. Confocal Microscopy and Image Analysis

Two dimensional (2D, xy) confocal images were recorded using a 20 × 1.0 numerical aperture air-immersion lens in the inverted configuration allowing excitation of rhod-2 at 543 nm, with emission collected above 550 nm with a long pass filter. For the mitochondrial uptake experiments time series x-y images (512 × 512 pixels, 0.5 ms/pixel) were taken at rest, following the first and fifth tetanus. To calculate rhod-2 fluorescence values originating from the mitochondria (F_mito_) a rectangle was drawn on the fiber, allowing the Zeiss Zen Blue (Zeiss, Oberkochen, Germany) image analyzer to calculate the average fluorescent values. The fluorescence was calculated at the peaks (I-band fluorescence, representing mitochondria (F_I-band_)) and at troughs (A-band, fluorescence, baseline, F_A-band_). The normalized mitochondrial calcium uptake expressed as F_mito_ was calculated using the formula:

F_mito_ = (F_I-band_ − F_A-band_)/F_A-band_ (2)

For the patch clamp studies line-scan (2D, xt) confocal image recordings were synchronized via pClamp 11.0 (Molecular Devices, San Jose, CA, USA) with the application of 100 ms long individual or repetitive depolarizing square pulses. Our Zeiss Live confocal microscope used a time resolution of 0.5 ms per line and a spatial resolution of 0.24 μm/line. The intensity of each pixel was digitized at 12-bit depth. Voltage evoked Ca^2+^ transients were analyzed by an in-house custom-developed program taking into account the dissociation constant for rhod-2 (K_d(rhod-2)_ = 1.58 µM) and k_ON_ = 0.07 µM^−1^ ms^−1^ as determined by Royer et al. (2008) [53]. The program calculated the baseline fluorescence (F_0_[x]) by averaging between 15 and 20 lines in the time domain prior to the first depolarizing pulse. Afterwards, the fluorescence intensity was expressed as normalized to F_0_[x] (F[x]/F_0_[x]). Ca^2+^ release flux was derived from cytosolic calcium transients ([Ca^2+^]_c_) using the removal method [54,55] in which only three removal processes must be considered: Binding to the monitoring dye, binding to EGTA and movement into the SR. From release flux, which is the flux calculated to leave exclusively through release channels, the net flux leaving the SR is derived by subtraction of the pump-removal flux. The difference between release and net flux, which is proportional to [Ca^2+^]_c_, is almost negligible due to the highly buffered environment achieved via EGTA. The integral of the net flux, from the beginning of the pulse until time t, defines the amount released at time t.

### 2.5. Molecular Biology

#### 2.5.1. RNA Preparation, Reverse Transcription (RT) and Quantitative Polymerase Chain Reaction (qPCR)

Total RNA fraction was isolated with TRI reagent (MRC, Cincinnati, OH, USA, cat. no.: TR118) from homogenized TA skeletal muscle specimens originated from CTRL, AX, and krill oil fed mice. The isolated RNA was resuspended in nuclease-free water (NFW) and stored at −80°C temperature. The RNA concentration and quality were determined by a spectrophotometer at 260 nm wavelength. (NanoDrop ND1000; Promega Biosciences, Madison, WI, USA). The isolated RNA was treated with DNase and RNase inhibitor (Ambion, Austin, TX, USA).

With the Omniscript RT kit (Qiagen, Germantown, MD, USA; cat. no.: 205113), 1000 ng of the isolated total RNAs were reverse transcribed into complementary DNA (cDNA); cDNA synthesis was done using random hexamers in 25 µL reaction volume.

For quantitative RT-PCR, Taqman Gene Expression Assays were used with the Taqman™ Gene Expression Master Mix (Applied Biosystems, Foster City, CA, USA). The amplification was performed using a Light Cycler 480 Master instrument (Roche, Basel, Switzerland) (cat. no. for plates, Roche: 04729692001; cat. no. for sealing foils, Roche: 04729757001).

Mouse Taqman gene expression assays were purchased from Thermo Fisher Scientific (Waltham, MA, USA), MCU (Mm01168773_m1), Mfn2 (Mm00500120_m1), CB1R (Mm01212171_s1), and Dnm1 (Mm01342903_m1). The amplification program was 10 min at 95 °C, followed by 50 cycles of 15 s at 95 °C and 1 min at 60 °C. The relative expression values for each transcript of interest were calculated by the comparative Ct method and AP3D1 (Mm00475961_m1) was used for normalization.

All qPCR reactions were performed in triplicates. Cp values were assessed with the Light Cycler 480 SW 1.5.0 software (Roche) and the relative copy numbers were calculated using the ∆Cp method. Ultimately, for the examined (MCU, Mfn2, CB1, Drp1) and normalization gene (AP3D1) the ratios of the measured values provided the relative expression levels.

#### 2.5.2. Western Blot

For Western blot experiments total cell lysates were isolated from the glutealis muscle by mechanical force extraction methods. Cell lysates from tissue samples were prepared with destruction of the cells by stainless steel balls. Protein content was measured by a modified BCA protein assay (Pierce, Rockford, IL, USA). The samples were then subjected to sodium dodecyl sulfate-polyacrylamide gel electrophoresis; 10% gels were loaded with equal (40 μg) amounts of protein per lane. Samples were then transferred to nitrocellulose membranes (Bio-Rad). The protein-binding nitrocellulose membranes were blocked with 5% dry milk-PBS solution. Proteins were probed with rabbit-anti-MICU1 primary antibodies (Thermo Fisher Scientific, Waltham, MA, USA, PA5-77364, polyclonal, 1:500), rabbit-anti-Mitofusin-2 (Cell signaling Technology, Danvers, MA, USA; 9482 and 8570, 1:500), and mouse-anti-Drp1 (Santa Cruz, Dallas, TX, USA, sc-271583, monoclonal, 1:500) with dilution in 5% milk containing PBS. As secondary antibodies, horseradish peroxidase-conjugated, goat anti rabbit IgG Fc segments (Bio-Rad; 1:1000 in 5% milk containing PBS) were used and the immunoreactive bands were visualized by a SuperSignal^®^ West Pico or Femto Chemiluminescent Substrate enhanced chemiluminescence kit (Pierce, Rockford, IL, USA) using a KODAK Gel Logic 1500 Imaging System (Eastman Kodak Company, Kodak, Tokyo, Japan). To assess equal loading, membranes were re-probed with mouse-anti-α-actinin antibody (1:1000 dilution in 5% milk containing PBS, Santa Cruz, Dallas, TX, USA, sc-166524) and visualized as described above.

### 2.6. Chemicals and Solutions

The composition of Krebs, patch internal, patch external, Tyrode’s solutions are detailed in our earlier report [39]. Fluorescent dyes (rhod-2 tripotassium salt and AM) were purchased from Invitrogen (Thermo Fischer, Waltham, MA, USA). All other chemicals were purchased from Sigma-Aldrich (St. Louis, MO, USA). Astaxanthin from *Haematoccocus pluvialis* (AstaReal A1010) was a kind gift for research purposes from AstaReal Sweden Co Ltd. (Nacka, Sweden) supplied in a 10 g vacuum package in an airtight aluminum bag carefully protected from air, heat and light. Krill oil from *Euphausia Superba* was a kind gift for research purposes from Rimfrost NZ Limited (Ålesund, Norway) supplied as a 200 g bottle.

### 2.7. Statistical Analysis

In this work the pooled data was expressed as mean ± standard error of the mean (SEM). For the in vivo grip test measurements paired *t*-test was employed to compare the data before and after the feeding with nutraceuticals for each group. For the in vitro force measurement, the mean and SEM were calculated as the averages of the values of muscles from the same animal, while the number of samples was the number of animals within the given group. We also employed one-way analysis of variance (ANOVA) and all pair wise Bonferroni’s multiple comparison method in order to compare the CTRL, AX and krill oil data. For all other cases the assessment of statistical significance was done using the statistical program Prism (GraphPad Software, San Diego, CA, USA).

## 3. Results

### 3.1. Increased Grip Force in Nutraceutical Supplemented Aging Mice

We have previously shown the beneficial effects of AX on body weight management and in vivo force in young mice [39]. Here we present similar results for both AX and krill oil supplemented aging mice. While AX administration significantly decreased the body weight by the end of four weeks on special diet, the krill oil supplemented animal group displayed similar weight management tendencies as those observed in the CTRL group (Table 1). The change in body weight was neither due to decreased food intake (the average consumption was 0.21 ± 0.01, 0.22 ± 0.02 and 0.21 ± 0.03 g/day/g body weight for CTRL, AX and krill oil, respectively) nor due to decreased muscle weight (for EDL see last line in Table 2; from *m. soleus* the values were: 17.04 ± 0.58 mg, 16.49 ± 0.81 mg (*p* > 0.4), 18.62 ± 0.95 mg (*p* > 0.1) for CTRL, AX and krill oil, respectively). To evaluate the in vivo muscle performance of the animals, grip tests were employed at the beginning and the end of the special diet period individually for each mouse. Both supplementations significantly increased the grip force normalized to the body weight after four weeks (Table 1).

### 3.2. Increased Twitch and Tetanic Force in EDL Muscles from Nutraceutical Treated Mice

**Figure 1:**
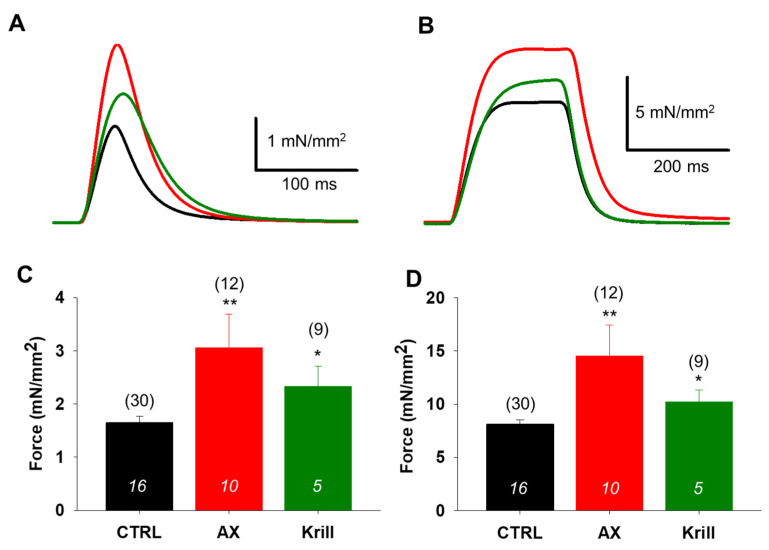
Increased EDL muscle force production in aging mice following AX and krill oil supplementation. Representative ex vivo twitch (**A**) and tetanic (**B**) force on EDL muscle from CTRL (black), AX (red) and krill oil (green) fed mice stimulated at 0.5 or 200 Hz, respectively, at room temperature (23–25 °C). The force was normalized to the cross-sectional area of the muscle. Averaged peak force of twitches (**C**) and tetani (**D**). The numbers in parenthesis indicate the number of muscles studied. The numbers in columns (italics) represent the number of animals used. * and ** denote significant difference from control at *p* < 0.05 and *p* < 0.01, respectively.

Positive effects of AX supplementation on in vitro force have been described previously in young mice [39]. Thus, we aimed in this study to measure twitch and tetanic force in EDL muscles obtained from aging mice. There was an increase in the amplitude of the normalized single twitches (Figure 1A) and tetani (Figure 1B) in AX and krill oil supplemented animals. The statistical analysis (paired *t*-test) proved significantly higher values concerning average peak twitch (Figure 1C) and tetanic (Figure 1D) force in both nutraceutical-treated groups compared to the control animals. However, our results point towards AX supplementation as being more potent, causing higher increase in the peak force. We did not detect significant differences between the measured parameters such as time to peak, half relaxation time of force and weight of EDL muscles in CTRL and treated mice (Table 2).

### 3.3. Minor Effects on Excitation-Contraction Coupling Mechanism Following Nutraceutical Administration

**Figure 2:**
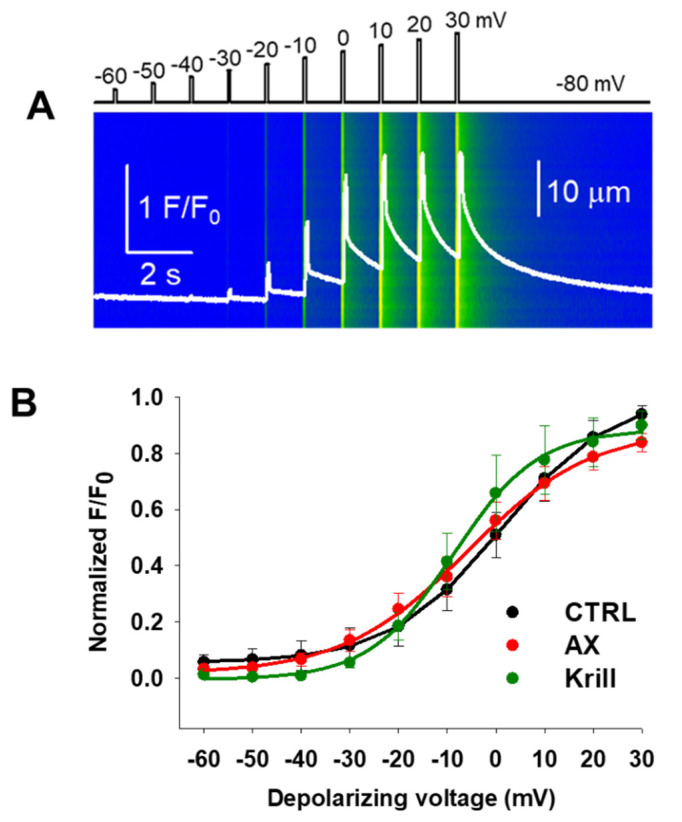
Nutraceutical administration leaves the excitation-contraction coupling mechanism of FDB fibers in aging mice unaltered. (**A**) Representative line-scan image of rhod-2 fluorescence normalized to the baseline value F_0_(x) in a control cell exposed to a series of subsequent square-shape depolarizing voltages (shown on top) using whole-cell voltage-clamp. The holding potential was −80 mV and the calcium transients were evoked by 100 ms long membrane depolarizations ranging from −60 mV to +30 mV, with 10 mV accrual. The delay between two pulses was 1100 ms. The white trace is the temporal profile of the normalized fluorescence obtained by averaging 50 lines in the spatial domain and normalized to the average resting F_0_(x) values. (**B**) Voltage dependence curves of the calcium transients. The normalized F/F_0_ values were fitted with a Boltzmann function and then normalized to the obtained maximum for a given fiber. The average values were plotted for individual fibers in each group. The continuous lines represent the best fit of the Boltzmann function to the average values with the following parameters: V_50_ = 1.07, −5.80 and −8.77 mV and k = 11.35, 12.78 and 8.65 for CTRL (*n* = 13 fibers, *N* = 7 mice), AX (*n* = 10 fibers, *N* = 5 mice), and krill oil (*n* = 6 fibers, *N* = 5 mice), respectively.

To assess whether four weeks of AX and krill oil treatment regimen had any effects on the excitation-contraction coupling of skeletal muscles, we examined calcium transients on single isolated FDB muscle cells using confocal microscopy conjugated with the whole-cell voltage clamp technique. While maintaining the holding potential at −80 mV, we applied 100 ms long square-shaped depolarization pulses between −60 mV and +30 mV, with 10 mV interval steps. Figure 2A shows a representative line-scan image recorded on a CTRL FDB fiber illustrating the applied protocol. Figure 2B shows the comparison of the normalized fluorescence from 13 CTRL (black), 10 AX (red), and 6 krill oil (green) treated fibers obtained from independent experiments like those shown in Figure 2A. The peaks of normalized F/F_0_ value for each depolarizing pulse were fitted with a Boltzmann function (see Equation (1) in *Materials and Methods*), then normalized to the obtained the maximum for the given fiber and ultimately averaged over the total number of fibers in each group (Table 3). By fitting a Boltzmann function to the averaged and normalized F/F_0_ values, the voltage dependence of calcium release can be characterized by the V_50_ (half activation voltage) and *k* values (slope of the Boltzmann curve). Based on the analysis of these parameters, we confirm no significant alterations in the release channel sensibility to voltage activation, although a slight non-significant negative shift was detected following nutraceutical diet.

### 3.4. Unaltered SR Calcium Transients and Fluxes by Nutraceutical Diet Regimen

**Figure 3:**
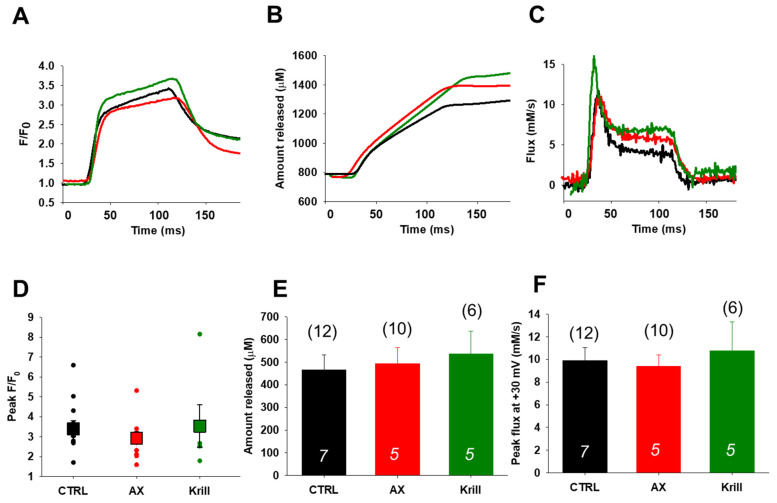
Comparison of the effects of nutraceuticals on Ca^2+^ transients and release fluxes in response to voltage clamp depolarizations in FDB muscle fibers of aging mice. (**A**) Fluorescence transients in response to 100 ms long depolarizing pulses to +30 mV. Traces in black, red, and green are for CTRL, AX, and krill oil, respectively. (**B**) Net amount of Ca^2+^ released, calculated for the transients in A. (**C**) Representative release flux traces calculated from the records shown in panel B. Note that no significant differences were observed following four weeks of nutraceutical diet regimen. (**D**) Pooled data for peak F/F_0_ values obtained at maximal depolarizing pulses (100 ms, +30 mV). The dots correspond to individual cells; the squares indicate the average (+SEM) calculated for each group. (**E**,**F**) Average (±SEM) data for peak released flux values and the integrals showing no significant changes. The numbers in columns (italics) indicate the number of animals studied, whereas the numbers in parenthesis denote the number of cells studied. All data sets were analyzed by one-way ANOVA.

Figure 3A, compares rhod-2 fluorescence transients recorded in CTRL (black), AX (red), and krill oil (green) FDB muscle fibers in response to fully activating depolarizing pulses (100 ms, +30 mV) under patch-clamp conditions. Figure 3B displays the net amount of Ca^2+^ released, whereas Figure 3C illustrates the release fluxes derived from the changes in intracellular [Ca^2+^]. No significant alterations of the mean peak fluorescence F/F_0_ values measured at maximal depolarizing pulses (+30 mV) were observed within each studied group (3.40 ± 0.39; 2.93 ± 0.31 and 3.53 ± 1.06 for CTRL, AX, and krill oil, respectively) (Figure 3D). Altogether, the traces (Figure 3B,C) and the pooled data (Figure 3E,F) show very similar trends in the CTRL and nutraceutical treated animals suggesting no major alterations of the SR calcium transients and release fluxes upon special diet utilization.

### 3.5. Preserved Activity Dependent Mitochondrial Calcium Uptake Following Nutraceutical Application

**Figure 4:**
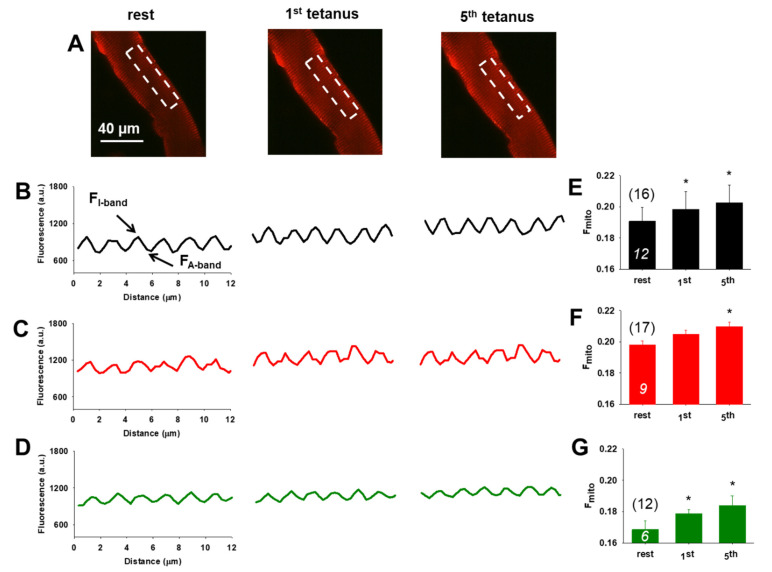
Stimulation-dependent changes in mitochondrial calcium levels in single FDB fibers from aging mice following four weeks of nutraceutical supplementation. (**A**) Illustration of rhod-2 AM fluorescence in a krill oil supplemented FDB fiber at rest and following tetanic stimulation (1 and 5 tetani, respectively). (**B**–**D**) Representative mitochondrial fluorescence intensity profiles plotted from similar highlighted square areas as depicted in images from A. (**E**–**G**) Average (±SEM) change of normalized mitochondrial fluorescence (F_mito_) calculated as (F_I-band_ − F_A-band_)/F_A-band_ for each group at rest and following a single or repetitive tetanic stimulation. The numbers in parenthesis denote the number of cells studied in each group. The numbers of animals are in columns (italics) for CTRL, AX, and krill oil, respectively. Note the tendency of activity-dependent mitochondrial calcium accumulation within each studied group; however no significant changes of the normalized F_mito_ values were observed between the CTRL and nutraceutical supplemented groups. * denote significant difference within the given group following tetanic stimulation at *p* < 0.05. All data sets were analyzed by one-way ANOVA.

Figure 4A illustrate representative confocal images recorded at rest, after the first, and the fifth tetanic stimulation of a single FDB muscle fiber incubated with rhod-2-AM. Following 500 ms long tetani at close to supramaximal voltage we asserted the fluorescence averaged over the spatial domain (white rectangle) and show the activity dependent triadic mitochondrial rhod-2 fluorescence increase as depicted by the traces in Figure 4B–D. The normalized mitochondrial fluorescence (F_mito_) evaluation was done similarly as in our earlier report [39]. Based on Equation (2) (see *Materials and Methods*) F_mito_ can be expressed as the differences between the peaks (I band fluorescence, corresponding to mitochondria) and troughs (A band fluorescence, corresponding to non-mitochondria or baseline). Figure 4E–G show the pooled mitochondrial fluorescence data for CTRL, AX, and krill oil supplemented groups, confirming the activity dependent mitochondrial accumulation starting from the resting state and immediately following the tetanic stimulation (0.192 ± 0.003, 0.198 ± 0.006 *, 0.202 ± 0.003 * for CTRL, 0.199 ± 0.002, 0.204 ± 0.002, 0.208 ± 0.002 * for AX and 0.168 ± 0.005, 0.178 ± 0.005 *, 0.183 ± 0.006 * for krill oil, respectively, with * *p* < 0.05 vs. rest). While as expected, F_mito_ shows an increasing tendency in each group, no significant changes were determined between the CTRL and nutraceutical supplemented groups.

### 3.6. Variable Effects of Nutraceuticals on Mitochondrial Dynamics

**Figure 5:**
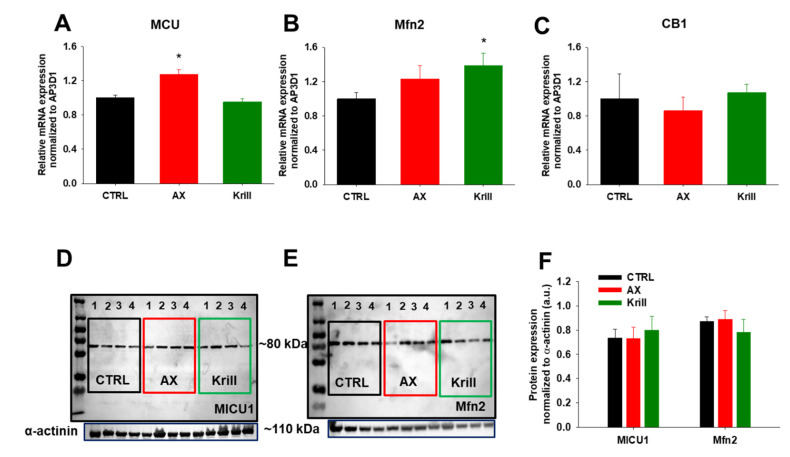
Nutraceutical administration has variable effects on the major proteins involved in mitochondrial bioenergetics. (**A**–**C**) RT-qPCR analysis shows significant differences in the levels of MCU and Mfn2 mRNA transcript expression in TA muscles of AX and krill oil supplemented mice. No differences were found in the levels of CB1 mRNA levels. Data were obtained from four mice/group. All results are shown as means ± SEM with * *p* < 0.05. (**D**,**E**) Representative Western blots against MICU1 and Mfn2, two key proteins involved in mitochondrial calcium homeostasis. The numbers on the gels indicate the number of animals studied. (**F**) Quantification of expression levels of MICU1 and Mfn2 proteins show no changes when normalized to α-actinin used as an internal control. Data (+SEM) are from two independent experiments.

Next, we were interested to elucidate whether the main proteins involved in mitochondrial dynamics (fusion and fission events) and Ca^2+^ regulation (Mfn2 and CB1 as these are known to be altered in aging) are somewhat affected by our nutraceutical diet regimen. For this, we performed quantitative PCR analysis on TA muscles (fast type II muscle fibers) and we found variable changes in the transcript levels of MCU, Mfn2 and CB1 following nutraceutical application. AX supplementation significantly increased the mRNA transcript level of MCU (Figure 5A), whereas krill oil had no influence on it. On the other hand, mRNA levels of Mfn2 were significantly altered by the latter, but only a mild increase was detected following AX supplementation (Figure 5B). Mitochondrial fission is a multi-step process that depends mainly on the cytosolic GTPase dynamin-related protein 1 (Drp1). Following AX but not krill oil supplementation our qPCR measurements revealed significantly increased Drp1 transcript levels, whereas on protein level a significant decrease was detected compared to the untreated group (Supplementary Figure S2). CB1 transcripts were unaltered following four weeks of nutraceutical diet (Figure 5C). These results possibly suggest that the increased Mfn2 and MCU transcript expression in aging muscles may be a consequence of changes in protein turnover. Thus, we examined by semi-qualitative Western blot the associated protein levels and found no significant changes upon nutraceutical utilization when compared to age-matched CTRL (Figure 5D–F). We concluded that four weeks of nutraceutical diet does not induce noteworthy alterations of mitochondrial dynamics and calcium signaling as determined by semi-quantitative means of protein level measurement.

### 3.7. Enhanced Spatial Learning and Memory in Aging Mice upon Krill Oil Supplementation

**Figure 6:**
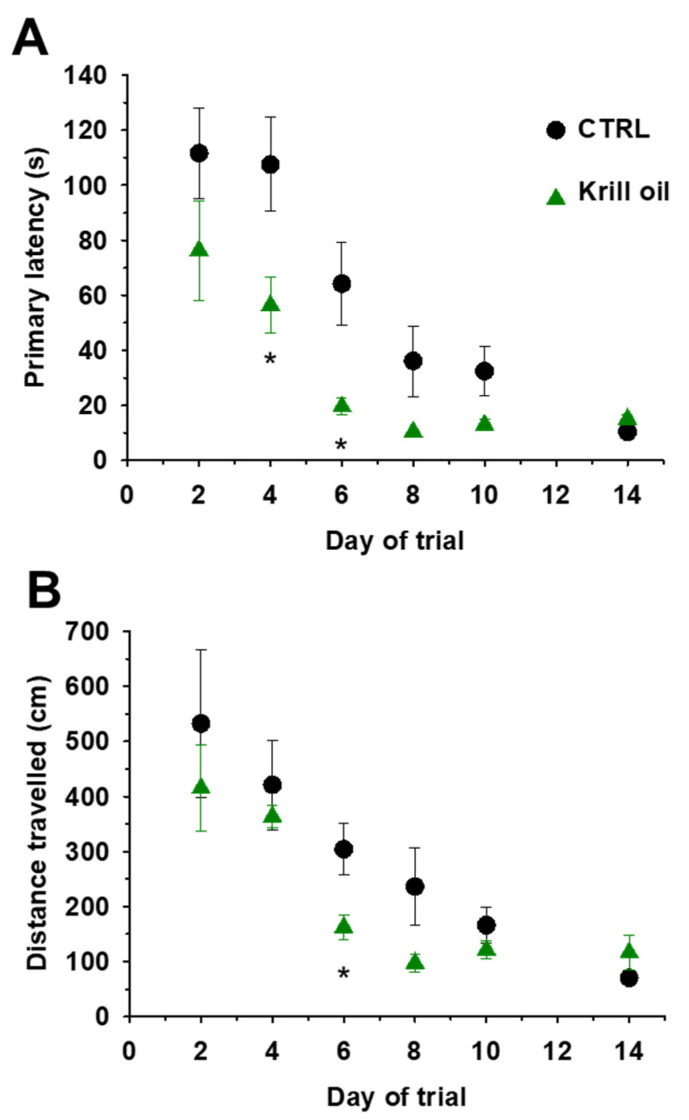
Krill oil supplementation enhances the spatial learning and memory in aging mice. (**A**) The time to find the escape tunnel location was improved by krill oil diet regimen. (**B**) Similarly, the length of the path to find the escape tunnel location as determined via the Barnes-Maze procedure was shorter in the krill oil supplemented group. Data points ± SEM were averaged every two days. * denotes significant difference between control and krill oil at *p* < 0.05.

To study the effects of n-3 PUFAs that are present in krill oil but not in AX, we employed the Barnes–Maze experiment on control and krill oil fed mice. Over the course of repeated trials, the observed differences (if any) in the latency (duration) and distance (path) covered to the escape hole can be interpreted as an indicator of hippocampus-dependent memory function. Eight CTRL and eight krill oil treated mice were trained in the Barnes–Maze for 10 consecutive days and after three days’ resting period the final test was conducted. During the training days, the latency in entering the target hole (primary latency) and the distance travelled to it was measured. During the training period the mice were getting familiarized with the position of the escape tunnel and day-by-day spent less time (Figure 6A) and covered shorter distance finding the target hole (Figure 6B). The starting average time to the escape box and the covered distance was essentially same in both animal groups. Krill oil supplemented mice learned the right position of the escape tunnel quicker and they were more purposeful than control animals (Supplementary Figure S1 and Videos S1 and S2). A significant difference (*p* < 0.05) was found between the two animal groups during the learning period in latency (days 4–6) and distance (day 6). It must be acknowledged that the final average value of each measured parameter was the same in both animal groups.

## 4. Discussion

### 4.1. Nutraceuticals and Skeletal Muscle Performance

Skeletal muscle produces ROS even at rest but more importantly during contractile activity causing oxidative stress and redox imbalances that can significantly contribute to fatigability, muscle weakness, reduction of contractile force and higher propensity to injuries especially in the elderly [56]. The idea that dietary supplementation with antioxidants such as vitamins C, E, A, and lately carotenoids such as astaxanthin can potentially alleviate oxidative damage, improve performance and promote optimal health has emerged [39,57,58]. The effects of AX on muscle strength and performance are being evaluated by many research groups (for a recent review see Wong et al. (2020) [59] and Sztretye et al. (2019) [36]). Lately, our workgroup has been interested in elucidating the aspects regarding the interaction of AX and calcium signaling in skeletal muscle. In our recent work [39] we propose a possible mode of action of AX in skeletal muscle: AX acts on the insulin receptor substrate (IRS) activating the PI3K/Akt pathway which induces GLUT4 translocation into the sarcolemma resulting in enhanced glucose uptake by muscle and ultimately leading to increased glycolysis (Figure 6 of the article cited above). Krill oil has been associated with improvement of exercise and antioxidant/anti-inflammatory markers and several clinical trials have been carried out. A recent review by Colletti et al. 2021 [60] details in great depth the aspects of krill oil actions on muscle performance and strength. One of the possible pathways is that krill oil activates mTOR signaling [61]. That is, a combination of EPA and DHA was found to increase the rates of muscle protein synthesis via an increase in activation of the mTOR-p70s6k signaling pathway in young- and middle-aged men and women [62]. Fish oil supplementation in combination with [63] or without [64] resistance exercise resulted in increased strength and functional ability in older adults.

In the present study we demonstrate that four weeks of two types of AX containing nutraceutical diet regimen significantly improved grip force in vivo (Table 1) and muscle force in vitro (Figure 1). These alterations occurred in parallel with a slight decrease of whole-body mass (Table 1) despite the virtually identical food intake for each animal group. One must note that the decreased body mass did not occur due to loss of muscle mass as EDL muscle weights were preserved during nutraceutical diet regimen (Table 2). One possible explanation for this increased muscle performance could be the accelerated lipid utilization paired with increased glucose uptake, thus an overall accelerated retinoic acid metabolic activity of the skeletal muscle tissue [65]. Furthermore, we cannot exclude the possibility that AX supplementation hinders the phosphorylation of ATP via MICU1 and shifts the energy consumption of skeletal muscle to more fatty acid oxidation, resulting in smaller body weight gain (Table 1). The authors can only hypothesize on this aspect as the study of the metabolic events were not the scope of this work and thus were not explored here.

One limitation of the present study is that the antioxidant status and overall total antioxidant capacity of muscle tissues were not evaluated here. Gao et al. (2020) [66] evaluated the effects of dietary supplementation of natural astaxanthin from *Haematococcus pluvialis* on antioxidant capacity, lipid metabolism and accumulation in the egg yolk of laying hens and found that the total antioxidant capacity, superoxide dismutase level and glutathione peroxidase level in the plasma, livers and egg yolks were significantly increased in the AX fed group. In mice, the review by Yoshihara et al. (2019) [67] gives a good summary on the effects of AX supplementation on skeletal muscle antioxidant capacity.

On the other hand, another possible explanation for the increased muscle force production upon nutraceutical administration could be the alteration of the calcium sensitivity of the contractile filaments and/or ryanodine receptor 1 (RyR1) release channel activity. As a consequence, the aspects pertaining to RyR1 function were explored next.

### 4.2. Nutraceuticals and Skeletal Muscle Calcium Handling

With aging ultrastructural changes develop in skeletal muscles, including the accumulation of tubular aggregates of SR membranes [68] that induce abnormal Ca^2+^ storage and release from the SR [69,70,71]. Additional molecular changes may involve quantitative (reduced expression) and functional modifications in the L-type voltage-dependent Ca^2+^ channels or dihydropyridine receptors (DHPRs) [72], which couple with the RyR1 to facilitate Ca^2+^ release from the SR after transverse tubules membrane depolarization via the ECC mechanism. In aged skeletal muscle fibers the functional uncoupling between DHPR and RyR1 leads to reduced calcium release from the SR [73]. RyR1s are very sensitive to oxidative changes and their function is also hindered in aging muscles because of oxidation and nitrosylation of their cysteine residues, resulting in Ca^2+^ leaks from the SR and causing muscle weakness [74].

Starting from the premises detailed above, we decided to examine by combining whole cell patch clamp and confocal imaging the possibility of altered coupling between the DHPRs and RyR1s in single FDB fibers from nutraceutical supplemented aging mice. Following four weeks of nutraceutical diet regimen we found no significant changes in the half activating voltages (Figure 2, Table 3) or peak calcium transients, release fluxes, and net amount of calcium released during 100 ms depolarizing pulses (Figure 3). Our results may suggest that the reduced calcium release previously described in aged mice [73] could be reversed/prevented via dietary nutraceutical supplementation in a voltage independent way.

### 4.3. Nutraceuticals and Mitochondrial Function and Antioxidant Features

There is common agreement on mitochondria having a role in Ca^2+^ regulation during ECC and contractile activity and that in aged mice the increased oxidative stress is paired with decreased mitochondrial Ca^2+^ uptake [75]. Here we explored the effects of nutraceuticals on stimulation induced mitochondrial Ca^2+^ uptake in rhod-2 AM loaded FDB fibers. While in young adult mice where AX supplementation favorably attenuated activity dependent mitochondrial calcium unbalance by downregulating mitochondrial calcium [39], in this work (performed on aging mice) these favorable effects were not observed (Figure 4). This could mean that both AX and krill oil due to their antioxidant actions lowered ROS production in FDB muscles which in turn could be associated with decreased RyR1 activity. The latter would manifest in smaller depolarization-induced calcium transients seen under voltage clamp; however, our data does not point out a dramatic change in calcium transients and release flux parameters. Thus, other processes might be at work; a possible explanation for the above might lay in the length of nutraceutical diet regimen application. Perhaps four weeks of special diet was sufficient to see beneficial effects in young adult mice but was insufficient in aging mice where oxidative stress and ROS production are more prominent. We hypothesize that the duration of nutraceutical feeding should be extended over a longer period; however, these events shall be explored in a future study.

Several defects were described in mitochondria isolated from skeletal muscles originating from old mice [76]. Cannabinoid receptor 1 was described to be abundantly present in striated muscle mitochondria where it controls mitochondrial oxidative activity [20,21]. It emerged that the activation of mtCB1 receptors in striated muscle may participate in the regulation of the oxidative activity of the muscle tissue. Ainbinder and colleagues [12] found that Mfn2 protein expression is progressively reduced in skeletal muscles and this marked reduction was not a consequence of reduced gene expression or lower availability of Mfn2 mRNA for translation. Similarly, in an elegant study Filadi and colleagues [77] found that cells lacking Mfn2 present decreased MCU expression levels which may be a reasonable explanation for the reduced mitochondrial Ca^2+^ signals in their experiments. This led us to explore whether nutraceutical treatment had any effect on the expression level of the key proteins involved in mitochondrial dynamics and proper calcium handling. As such, first we examined the relative mRNA transcript levels of Mfn2, MCU, Drp1, and CB1 via qRT-PCR reaction and found variable effects following nutraceutical diet (Figure 5A–C, Supplementary Figure 2A); most importantly when quantifying the associated protein levels the above differences were not detectable (Figure 5F). Thus, we conclude that the unaltered Mfn2 and MICU1 protein levels detected by Western blot (Figure 5D–F) may explain the preserved parameters of the calcium transients and mitochondrial Ca^2+^ signals seen during our patch clamp and repetitive stimulation induced Ca^2+^ uptake experiments.

### 4.4. Krill Oil and Cognition

Krill oil used in our work was supplied by Rimfrost Inc (Alesund, Norway). According to the company website: “Rimfrost Krill Oil is gently produced with a unique patented extraction technology from sustainably harvested and 100% traceable Antarctic krill, into pure, high quality krill oil with no additives. The krill oil is a rich and highly bioavailable source of the marine omegaΩ fatty acids DHA/EPA in the form of phospholipids, meaning they are easily absorbed, boosting the body’s ability to use them. The unique extraction technology preserves 2–3 times more of the natural astaxanthin in the krill than other krill oils on the market. This high level of astaxanthin helps prevent oxidation, stabilizing the products and extending the shelf life”. In summary, krill oil contains a high level of n-3 PUFAs and phospholipids (PLs) and minor components such as vitamins, minerals, astaxanthin and flavonoids. It is an excellent nutritional source of the essential nutrient choline and a broad range of various amino acids including essential amino acids. The content of the different classes of nutritional components is greatly influenced by the extraction technologies [60,78]. The exact percentage of each constituent was not disclosed to us by Rimfrost Inc. as that is patented information [79]. Krill oil contains approximately 0.5 mg of astaxanthin per 3 g of krill oil, which is below the currently established effective dose of 4 mg for athletes [60]. In our current experiments we used 25 g/kg of krill oil in order to achieve a similar AX concentration of 0.02% as in our previous work [39].

Age is considered a crucial factor in cognition, characterized by a progressive decline in spatial memory performance and highly influencing the morphology of brain structures involved in cognition processes. AX has been proposed as a presumed neuroprotective compound able to preserve brain aging [80]. The effects of AX supplementation on the brain were not directly evaluated here. We only asserted the effects of krill oil via the Barnes–Maze protocol. Others, however, did study the effects of AX on the brain [81]. Grimming and coworkers [40] evaluated the effect of AX on cognitive function and neural plasticity in young and elderly mice. The authors found that one month supplementation with AX improved cognitive performance and increased long-term potentiation in older mice. This is in line with our current observation that four weeks of krill oil supplementation exerted positive effects on spatial learning and memory in aging mice as assessed via the Barnes–Maze test. Our data clearly demonstrate improved cognitive capacity in aging mice in terms of faster learning (Figure 6, Supplementary Figure S1, Videos S1 and S2).

Fish oil and krill oil contain high levels of PUFAs (EPA and DHA) bound in different structural forms. In fish oil, EPA and DHA are found as triglycerides and thus are less bioactive than in krill oil where they are mainly in the form of PLs (in a 2:1 ratio) making them readily bioavailable especially for the brain that contains up to 60% lipids (a large fraction of this is PUFAs, predominantly DHA) [82]. There are contradictory data regarding the effects of fish oil supplementation in mice. de Magalhães and coworkers [83] concluded that fish oil supplementation does not extend longevity in normal healthy mice. A similar conclusion was reached by other groups who found that consumption of isolated n-3 fatty acid-rich oils will not increase the lifespan or health of initially healthy individuals but rather would increase oxidative stress, decrease cellular function and cause organ dysfunction [84,85]. On the other hand, krill oil was found to have beneficial effects and facilitate learning processes [86], provide antidepressant-like effects in rats without presenting sedative effects [87], and increase the amount of EPA and DHA in the brain of Zucker rats [88]. These results together with our findings suggest that the lipid composition of krill oil plays a determining role in its ability to improve cognitive functions in aging mice. However, further studies are needed to uncover whether AX alone would have any direct effects on the neurons of the CNS.

## 5. Conclusions

**Figure 7:**
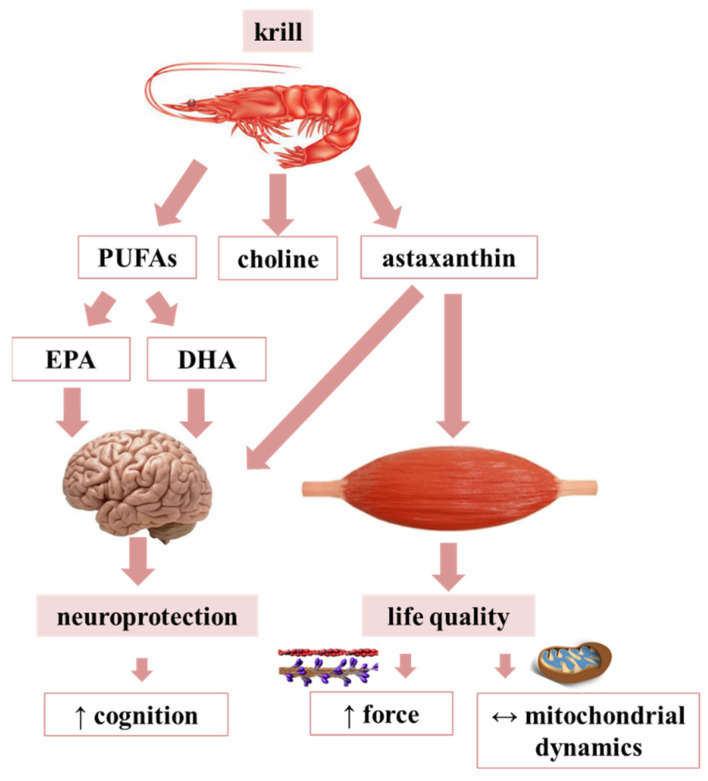
Summary on the mode of action of nutraceuticals in aging mice.

Aging leads to deficits in skeletal muscle function and strength in conjunction with impairment of cognitive capacities. With the escalation of life expectancy, it has become an essential target to preserve health and fitness and maintain proper brain function and overall well-being within the elderly population. Thus, tackling healthy aging and uncovering the changes in muscle fiber signaling that lead to impairment in whole muscle contractile properties along with deciphering the steps of calcium regulation are crucial tasks. Since one of the hallmarks of aging is a progressive loss of muscle mass, strength and locomotion in this study we explored the effects of two potentially geroprotector antioxidant agents (krill oil and AX) on alleviating oxidative stress, skeletal muscle performance and elements of calcium homeostasis with emphasis on the ECC mechanism and mitochondrial calcium regulation in aging mice. Based on our present data on aging mice as summarized in Figure 7, it seems that nutraceutical supplementation has beneficial effects on muscle performance and cognition without drastically changing ECC and mitochondrial calcium signaling. We believe that the PUFA content present in the PL form in krill oil is the major contributor to the improved cognitive function. However, we admit that direct evaluation of AX concentration in this respect has not been carried out in present work. We consider that such experiments, i.e., how AX directly influences the neurons of CNS, would increase our understanding on the overall effects of geroprotector nutraceutical(s) on human function and behavior in both the young and the elderly. Nevertheless, we propose that geroprotective nutraceuticals with strong antioxidant action (i.e., AX and krill oil) possibly complemented with other interventions such as exercise and hormonal and nutritional mediators may provide good efficacy in promoting brain health, skeletal muscle fitness and performance and may cease/reverse frailty during aging.

## Figures and Tables

**Table 1:** The change in body weight and grip force after the application of special nutraceutical diet.

Parameters	CTRL	AX	Krill Oil
Number of animals	6	4	7
Body weight change (g)	−1.04 ± 0.66	−3.98 ± 0.31 ***	−0.73 ± 0.34
Maximal force change (mN)	−6.45 ± 5.87	−1.46 ± 6.08	11.09 ± 5.33
Normalized force change (mN/g)	−0.11 ± 0.23	0.42 ± 0.23 *	0.44 ± 0.16 *

* and *** denote significant difference from 0 at *p* < 0.05 and 0.001, respectively.

**Table 2:** Contractile force parameters from EDL muscles of 16 CTRL, 10 AX, and 5 krill oil treated mice following nutraceutical application.

Parameters	Twitch			Tetanus		
	CTRL	AX	Krill Oil	CTRL	AX	Krill Oil
Number of muscles	30	12	9	30	12	9
Force (mN/mm^2^)	1.65 ±0.12	3.06 ± 0.63 **	2.33 ± 0.38	8.13 ± 0.40	14.52 ± 2.94 **	10.24 ± 1.11 *
TTP (ms)	30.1 ± 0.3	31.0 ± 1.3	34.3 ± 1.7 ***	173.7 ± 4.4	177.8 ± 7.8	190.1 ± 5.8
HRT (ms)	26.0 ± 0.6	26.0 ± 1.3	28.1 ± 1.4	80.2 ± 4.6	76.7 ± 8.3	65.7 ± 7.2
Muscle weight (mg)	17.6 ± 0.4	17.0 ± 0.9	18.6 ± 0.9			

*, ** and *** denote significant difference compared to control at *p* < 0.05, 0.01 and 0.001, respectively. Abbreviations: TTP—time to peak; HRT—half relaxation time.

**Table 3:** Individually averaged Boltzmann parameters from 7 CTRL, 5 AX, and 5 krill oil treated mice.

Parameters	CTRL	AX	Krill Oil
Number of muscle fibers	13	10	6
k	9.41 ± 0.74	12.54 ± 1.47	8.97 ± 1.56
V_50_ (mV)	−0.95 ± 4.82	−5.87 ± 3.89	−2.62 ± 5.86

## Data Availability

The raw data supporting the conclusions of this article are contained within the article.

## Supplementary Materials

The following are available online at https://www.mdpi.com/article/10.3390/antiox10091415/s1, Figure S1: Assessment of latency and distance covered to find the target hole on the 6th day of training of Barnes–Maze test following 4 weeks of krill oil supplementation. Figure S2: The effects of nutraceutical administration on mitochondrial fission protein Drp1. Videos S1 and S2: The movement of a CTRL and a krill oil treated mouse on the 6th day of Barnes–Maze training protocol (The Video S1 and S2 have been uploaded to the external site https://zenodo.org/deposit/5211540, accessed on 7 July 2021).
