# Supplementary figures and images for: Assessing the Potential of Nutraceuticals as Geroprotectors on Muscle Performance and Cognition in Aging Mice
Source: Antioxidants (Basel). 2021 Sep 4;10(9):1415. doi: 10.3390/antiox10091415 (PMC8472831; doi:10.3390/antiox10091415)

**CTRL**

**A**

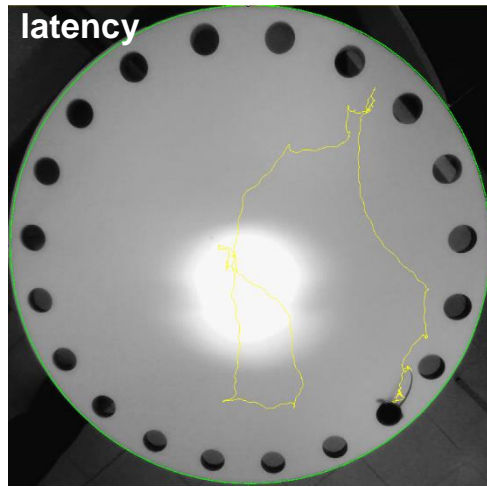

**Krill oil**

**C**

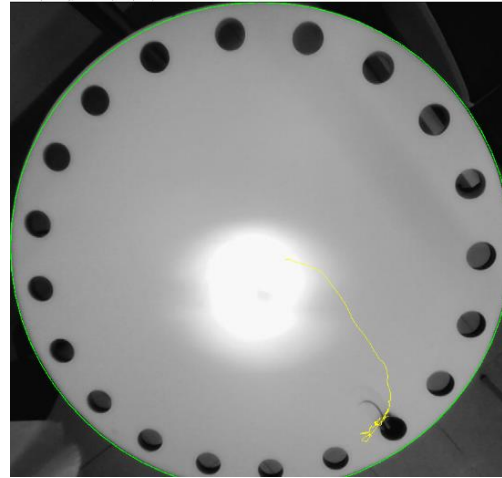

**B**

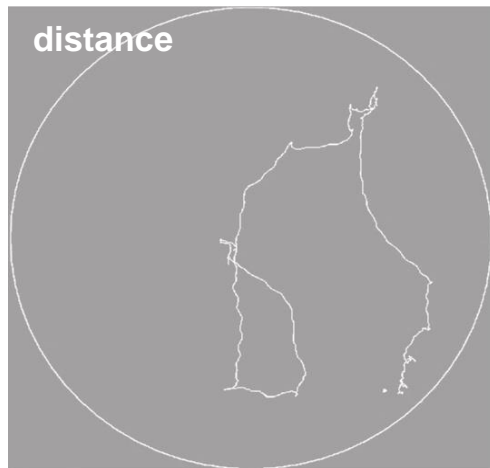

**D**

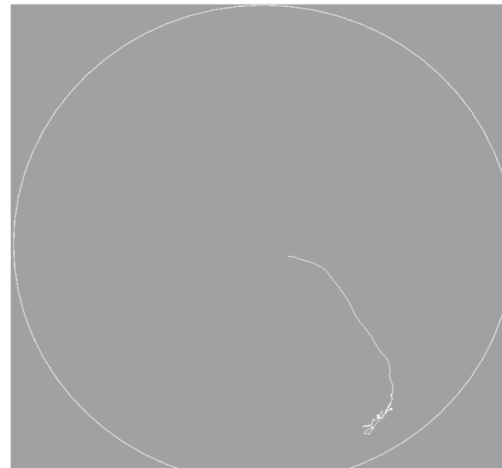

**A**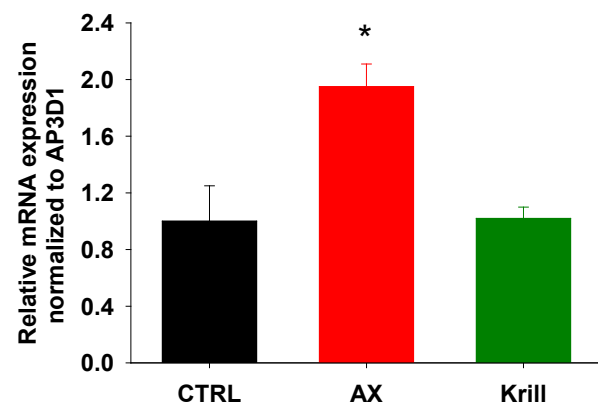**B**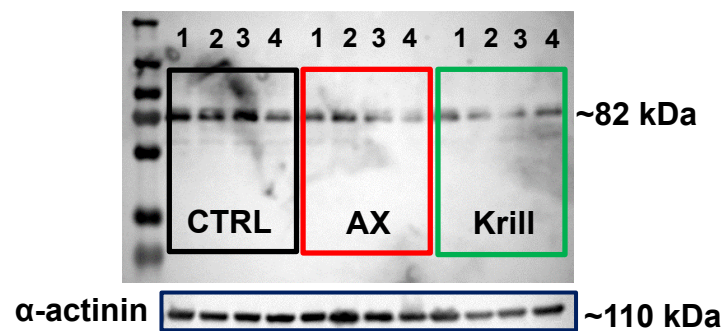**C**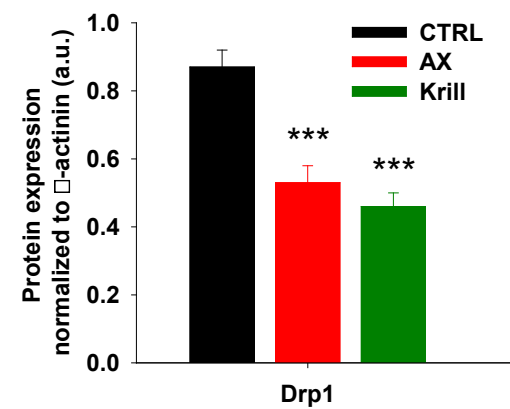

Supplement: Supplementary file 1 [file antioxidants-10-01415-s001.zip › antioxidants-1310968-supplementary.pdf]
